# Worksite health screening programs for predicting the development of Metabolic Syndrome in middle-aged employees: a five-year follow-up study

**DOI:** 10.1186/1471-2458-10-747

**Published:** 2010-12-02

**Authors:** Yu-Cheng Lin, Jong-Dar Chen, Su-Huey Lo, Pau-Chung Chen

**Affiliations:** 1 Health Management Center, Tao-Yuan General Hospital, Tao-Yuan, Taiwan; 2 Institute of Occupational Medicine and Industrial Hygiene, College of Public Health, National Taiwan University, Taipei, Taiwan; 3 School of Medicine, Fu Jen Catholic University, Taipei, Taiwan; 4 Department of Family Medicine, Shin Kong Wu Ho-Su Memorial Hospital, Taipei, Taiwan; 5 Department of Internal Medicine, Tao-Yuan General Hospital, Tao-Yuan, Taiwan

## Abstract

**Background:**

Metabolic syndrome (MetS) management programs conventionally focus on the adults having MetS. However, risk assessment for MetS development is also important for many adults potentially at risk but do not yet fulfill MetS criteria at screening. Therefore, we conducted this follow-up study to explore whether initial screening records can be efficiently applied on the prediction of the MetS occurrence in healthy middle-aged employees.

**Methods:**

Utilizing health examination data, a five-year follow-up observational study was conducted for 1384 middle-aged Taiwanese employees not fulfilling MetS criteria. Data analyzed included: gender, age, MetS components, uric acid, insulin, liver enzymes, sonographic fatty liver, hepatovirus infections and lifestyle factors. Multivariate logistic regression was used to estimate the adjusted odds ratios (OR) and 95% confidence interval (CI) of risk for MetS development. The synergistic index (SI) values and their confidence intervals of risk factor combinations were calculated; and were used to estimate the interacting effects of coupling MetS components on MetS development.

**Results:**

Within five years, 13% (175 out of 1384) participants fulfilled MetS criteria. The ORs for MetS development among adults initially having one or two MetS components were 2.8 and 7.3, respectively (both p < 0.01), versus the adults having zero MetS component count at screening. Central obesity carried an OR of 7.5 (p < 0.01), which far exceeded other risk factors (all ORs < 2.7). Synergistic effects on MetS development existed between coupling MetS components: 1. High blood pressure plus low-HDL demonstrated an OR of 11.7 (p < 0.01) for MetS development and an SI of 4.7 (95% CI, 2.1-10.9). 2. High blood pressure plus hyperglycemia had an OR of 7.9 (p < 0.01), and an SI of 2.7 (95% CI, 1.2-6.4).

**Conclusion:**

MetS component count and combination can be used in predicting MetS development for participants potentially at risk. Worksite MetS screening programs simultaneously allow for finding out cases and for assessing risk of MetS development.

## Background

Metabolic syndrome (MetS) management programs conventionally focused on the adults having MetS [1-3]; while risk assessment for MetS development is also important for many middle-aged adults potentially at risk but do not yet fulfill MetS criteria at screening. Risk assessments for MetS development by utilizing data from lifestyle investigations [4,5] or MetS components, such as elevated blood pressure, dyslipidemia and obesity, have been studied in general population surveys [6,7]. In terms of multi-functional applications and budget effectiveness for health managements, it is worth to investigate whether the records, such as MetS component measurements, from a MetS screening program can be further applied on the risk assessment for MetS development. For the large middle-aged working population, however, there is a lack of comprehensive follow-up survey examining whether 1. The MetS component count, 2. Each individual MetS component or 3. The combinations of individual MetS components are associated with MetS development. Since periodic routine health examinations are compulsory for workers at many worksites in Taiwan, we have the opportunity to observe the development of MetS among middle-aged employees. Therefore, we conducted this workplace-based retrospective follow-up study to evaluate whether the readymade database from the MetS screening program is helpful in risk assessment for MetS development in middle-aged Taiwanese adults.

## Methods

### Study populations

In accordance with the *Labor Health Protection Regulation*, 1648 employees from an electricity manufacturing company underwent compulsory health checkups in 2002 and 2007. The final analysis of this follow-up study included only the adults who did not fulfill the MetS criteria in 2002. Among the 1648 workers undergoing health examinations in both 2002 and 2007, 256 employees' records were excluded from the present study because of having MetS in 2002. A total of 1384 adults (338 female and 996 male) constituted a cohort; and their records were used for endpoint analysis. This health examination was open to all registered employees during every working day, for one-month duration. The examinees were suggested to avoid strenuous physical exercise during the three-day span prior to undergoing the health examination. Subjects' identities were anonymous and unlinked to the data. This analytical study followed the ethical criteria for human research; the study protocol (TYGH09702108) was reviewed and approved by the Ethics Committee of the Tao-Yuan General Hospital, Taiwan. Written informed consent was obtained from each participant.

### Demographics, lifestyle data, biological measurements and sonogram examinations

In 2002, a questionnaire about baseline personal history, including physical exercise, smoking, drinking and dietary habits, was completed by the examinees. Physical examinations and blood tests were performed on all participants in both 2002 and 2007. The participants arrived at the health care unit of the factory in the morning between 07:30 and 09:30 AM, after an overnight 8 h fast. The physical examination records included measurements of waist circumference, weight, height, and blood pressure. Waist circumferences were measured midway between the lowest rib and the superior border of the iliac crest. After being seated for 5 min, sitting blood pressure was measured on the dominant arm using digital automatic sphygmomanometers (model HEM 907, Omron, Japan) two times at a 5 min interval; the mean of the reading was used for data analysis. After the physical examination, participants were placed in a reclining position, and venous blood (20 ml) from an antecubital vein of the arm was taken for subsequent tests. Blood specimens were centrifuged immediately thereafter; and shipped frozen in dry ice to central clinical laboratory (certified by ISO 15189 and ISO 17025) in Tao-Yuan General Hospital. Glucose, triglyceride, high-density lipoprotein (HDL) cholesterol, alanine aminotransferase (ALT), γ-glutamil transferase (γ-GT) and uric acid analyses were conducted by a Hitachi autoanalyzer, model 7150 (Hitachi, Tokyo, Japan). Hepatitis B virus (HBV) surface antigen (HBsAg) and anti-Hepatitis C virus (HCV) antibody were measured by the microparticle enzyme immunoassay using the Axsym System instrument (Abbott, Illinois, USA). Insulin was determined by radioimmunoassay using an Axsym autoanalyzer (Abbott, Illinois, USA).

Since sonographic diagnosis for fatty liver has acceptable agreement among operators[8] and is widely accepted in many epidemiological surveys[9], this noninvasive method was used to diagnose fatty liver. In 2002, abdominal ultrasound examinations were performed for all examinees using convex-type real-time electronic scanners (Toshiba SSA-340 with 3.75 MHz convex-type transducer).

### Definitions

We defined "having routine physical exercise" as answering, "Doing exercises more than three times every week." To define "ever been a smoker", the first question was "do you smoke? (1. Never, 2. Currently smoke, 3. Previously smoked, have since quit)," and this was followed by questions about frequency. We defined "ever been a smoker" as answering "yes" to question 2 or 3 and consuming at least 6 cigarettes daily for over one year. For defining "habitual drinker", the first question was "do you usually consume alcohol? (1. No, 2. Yes)," and this was followed by questions regarding frequency and quantity; we defined "habitual drinker" in this study as saying "yes" to previous question and drinking at least one time weekly, over one year, with alcohol consumption more than 40 g/day for males or 20 g/day for females[10]. For defining "used to having snacks", the first questions were "are you used to having snacks before sleeping?" and "are you used to having snacks between meals?" These were followed with questions regarding quantity. We defined snacking habits in the present study as more than three times per week. The standard portions of examples for snacks (fruit products, milk products, fried food, nuts, beans products, meat, and alternatives) were explained to examinees in questionnaires.

MetS was diagnosed if the examinees had three or more of the following components: central obesity (COB) was identified as a waist circumference > 90 cm for male and > 80 cm for female; high blood pressure (HBP) was defined as systolic blood pressure (SBP) ≥ 130 mmHg or diastolic blood pressure (DBP) ≥ 85 mmHg. Hyperglycemia (Hyper-GL), hypertriglyceridemia (Hyper-TG) and low-HDL cholesterolemia (Low-HDL) were defined as fasting sugar ≥ 100 mg/dL, triglycerides ≥ 150.mg/dL and HDL < 40 mg/dL for males and < 50 for females (Bureau of Health Promotion in Department of Health, Taiwan) [11,12]. We specified the adults having MetS outcomes as those who did not fulfill MetS criteria in 2002 but did in 2007. Hyperinsulinemia(> 11.6 μU/ml) was arbitrarily defined as the top quartile level of our analyzed population as in other studies [13,14]. Elevated ALT (> 40 U/L), γ-GT (> 50 U/L) and hyperuricemia (> 8 mg/dL) were defined as having a value greater than the normal range established by the Tao-Yuan General Hospital clinical laboratory [15].

Sonographic fatty liver was assessed using the following five parameters: liver-kidney contrast, gall bladder wall masking, blurring of wall of hepatic and portal veins, far attenuation of the diaphragm; and diagnosis was made according to previous reports [16].

### Statistical analysis

Baseline characteristics and abnormal rates were compared between subgroups with/without MetS outcome within five years using the *t*-test and the χ^2 ^test for categorical and continual variables as appropriate. Three models of multivariate logistic regression were used to estimate the adjusted odds ratio (OR) and 95% confidence intervals (CI) of risk factors for predicting MetS development, under the adjustments for age, gender, previously documented predictors of MetS development (hyperinsulinemia, hyperuricemia, elevated liver enzymes, and sonographic fatty liver), lifestyle, each MetS component, the MetS component count and their combinations. A two-sided P-value < 0.05 was considered statistically significant.

The synergistic index (SI) value of potential risk factors A and B was therefore calculated by following the mathematical formula. A value of SI > 1.0 indicated that the interaction was more than an additive interaction, i.e., synergism[17].

$$\text{SI}=\;\frac{\left[\text{OR}\;(\text{A}\;\text{plus}\;\text{B})-1\right]}{\left[\text{OR}\;\text{(A}\;\text{alone)}+\text{OR}\;\text{(alone)}\;-2\right]}$$

SAS version 8.0 (SAS Institute, Cary, NC, USA) was used for all statistical analyses.

## Results

Within a five-year interval, 13% (175 out of 1384) of the participants met the criteria of MetS. Table 1 summarizes the overall baseline characteristics and abnormality rates of the sample population in 2002. The initial mean age for this population was 32.3 years. Significant differences were found when grouping baseline data according to the MetS outcome: as shown in the upper rows of Table 1, the adults having MetS outcome had significantly unfavorable initial measurements including baseline body mass index, waist, blood pressures, blood sugar, lipid profiles, liver enzymes, uric acid and insulin. Also shown in the lower rows of Table 1, this was a male-dominated (72.0%) working population; there were 58.6% participants initially having at least one MetS components; 26.1% smoked; 7.2% regularly consumed alcohol at least once a week and 32.1% did exercise at least three times per week. There were 27.4% of the participants having fatty liver. HBV and HCV carriers were 18.5% and 1.2% of sample population, respectively. With respect to MetS outcome, the adults initially having MetS components, the males, the habitual drinkers and the adults having fatty liver significantly prone to have MetS outcome within five years (Table 1).

**Table 1:** Baseline characteristics for Taiwanese adult workers not fulfilling metabolic syndrome (MetS) criteria initially

		2002 baseline data, divided by MetS outcome in 2007**		
			
Variable*	Overall N = 1384	Without MetS outcome	With MetS outcome	*P*
		n = 1209	n = 175	
Age (years)	32.3 ± 6.5	32.1 ± 6.5	33.3 ± 6.6	0.04
Body mass index (kg/m^2^)	23.0 ± 3.0	22.6 ± 2.9	25.8 ± 2.5	<0.01
Waist (cm)	75.6 ± 8.6	74.4 ± 8.2	83.3 ± 7.4	<0.01
Systolic blood pressure (mmHg)	117.0 ± 14.3	116.0 ± 14.1	124.2 ± 14.2	<0.01
Diastolic blood pressure(mmHg)	71.7 ± 9.2	71.0 ± 9.0	76.4 ± 9.0	<0.01
Fasting blood sugar (mg/dl)	94.3 ± 13.2	93.7 ± 12.0	97.8 ± 19 6	<0.01
Triglyceride (mg/dl)	102.5 ± 78.0	97.3 ± 77.3	137.9 ± 73.3	<0.01
HDL cholesterol (mg/dl)	50.5 ± 11.8	51.2 ± 11.7	46.0 ± 12.0	<0.01
Alanine aminotransferase (U/l)	27.2 ± 26.6	25.5 ± 25.8	38.5 ± 29.3	<0.01
γ-Glutamyl transferase (U/l)	33.4 ± 24.7	31.0 ± 22.2	49.7 ± 33.3	<0.01
Uric acid (mg/dl)	6.6 ± 1.6	6.5 ± 1.6	7.2 ± 1.5	<0.01
Insulin (μU/ml)	8.2 ± 5.7	7.8 ± 5.0	11.1 ± 8.9	<0.01
				
Adults having MetS component (count ≤ 2)	811 (58.6%)	657 (54.3%)	154 (88.0%)	<0.01
Male gender	996 (72.0%)	855 (70.7%)	141(80.6%)	<0.01
Ever been a smoker (≥ 6 cigarettes/day for ≥1 year)	361 (26.1%)	312 (25.8%)	49 (28.0%)	0.55
Regular alcohol consumption (≥ 1 day/week)	100 (7.2%)	77 (6.4%)	23 (13.1%)	0.01
Taking exercise ≥ 3 days/week)	444 (32.1%)	389 (32.2%)	55 (31.4%)	0.84
Having snacks before sleeping (≥ 3 days/week)	644 (46.5%)	568 (47.0%)	76 (43.4%)	0.38
Having snacks between meals (≥ 3 days/week)	546 (39.5%)	473 (39.1%)	73 (41.7%)	0.51
HBV carrier	256 (18.5%)	221 (18.3%)	35 (20.0%)	0.59
HCV carrier	16 (1.2%)	13 (1.1%)	3 (1.7%)	0.53
Sonographic fatty liver	349 (27.4%)	278 (23.0%)	101 (57.7%)	<0.01

* The upper rows are means and standard deviation (mean ± SD), and the lower rows are proportions (numbers and proportions,%). Abbreviations: HDL cholesterol, high-density lipoprotein cholesterol.

** t-tests for continual variables between groups with/without MetS outcome; χ^2 ^tests were conducted for categorical variables between groups with/without MetS outcome

The initial prevalence rates of individual MetS components and their combinations are shown on Table 2. Upper rows demonstrate the prevalence of MetS components (not segregated from each other): COB, 3.9%; Hyper-GL, 20.5%; Low-HDL, 24.4%; HBP, 17.7%; and Hyper-TG, 13.7%. The initial abnormality rates of each component were significantly unfavorable for the adults having MetS outcome. Table 2 also shows that the baseline prevalence rate of single MetS component was 36.9%; and that 21.7% of sample population had two MetS components. Among the adults initially having only one MetS component, baseline distributions of each single MetS component were non-significantly different between the groups with/without MetS outcome (Table 2, middle rows). In contrast, the adults initially having two MetS components predominated significantly in the group having MetS outcome (Table 2, lower rows).

**Table 2:** Baseline prevalence rates for Taiwanese adult workers not fulfilling metabolic syndrome (MetS) criteria initially

		2002 baseline data, divided by MetS outcome in 2007**		
			
Abnormality Prevalence*	Overall N = 1384	Without MetS outcome n = 1209	With MetS outcome, n = 175	*P*
MetS component (not segregated)				
COB	54 (3.9%)	28 (2.3%)	26(14.9%)	<0.01
Hyper-GL	284 (20.5%)	234 (19.4%)	50(28.6%)	0.01
HBP	245 (17.7%)	193 (16.0%)	52(29.7%)	<0.01
Low-HDL	338 (24.4%)	269 (22.2%)	69(39.4%)	<0.01
Hyper-TG	190 (13.7%)	139 (11.5%)	51(29.1%)	<0.01

MetS component (segregated by count and combination)				
Single component	511 (36.9%)	451 (37.3%)	60 (34.3%)	0.44
Hyper-GL alone	149 (10.8%)	132 (10.9%)	17(9.7%)	0.62
Low-HDL alone	168 (12.1%)	151 (12.5%)	17(9.7%)	0.27
HBP alone	115 (8.3%)	107 (8.9%)	8(4.6%)	0.02
Hyper-TG alone	63 (4.6%)	51 (4.2%)	12(6.9%)	0.19
COB alone	16 (1.2%)	10 (0.8%)	6(3.4%)	0.07
Two components	300 (21.7%)	206 (17.0%)	94 (53.7%)	<0.01
Low-HDL-plus-Hyper-TG	61 (4.4%)	43 (3.6%)	18(10.3%)	<0.01
Hyper-GL-plus-Low-HDL	51 (3.7%)	42 (3.5%)	9(5.1%)	0.34
Low-HDL-plus-HBP	42 (3.0%)	26 (2.2%)	16(9.1%)	<0.01
Hyper-TG-plus-HBP	31 (2.2%)	22 (1.8%)	9(5.1%)	0.05
Hyper-GL-plus-Hyper-TG	30 (2.2%)	22 (1.8%)	8(4.6%)	0.09
COB-plus-Low-HDL	16 (1.2%)	7 (0.6%)	9(5.1%)	<0.01
COB-plus-HBP	10 (0.7%)	5 (0.4%)	5(2.9%)	0.06
COB-plus-Hyper-GL	7 (0.5%)	5 (0.4%)	2(1.1%)	0.38
COB-plus-Hyper-TG	5 (0.4%)	1 (0.1%)	4(2.3%)	0.05

* The abnormality prevalence is demonstrated as numbers and proportions, %. The upper rows are non-segregated distributions of MetS components, one or two risk factors are included and overlapping can happen; the lower rows are segregated by each signal and combinations, all individual conditions are listed. Abbreviations: Hyper-GL, hyperglycemia; Low-HDL, Low-HDL cholesterolemia; HBP, high blood pressure; Hyper-TG, hypertriglyceridemia; COB, central obesity.

**χ^2 ^tests were conducted for categorical variables between groups with/without MetS outcome.

As shown in Table 3, 4 and 5 we constructed three models for the multivariate analyses. Utilizing a conventional model, after controlling for confounders, Table 3 demonstrates that MetS components, sonographic fatty liver, elevated γ-GT and hyperinsulinemia were independent risk factors for MetS development among our early-middle-aged employees. Table 3 also indicates that the middle-aged adults getting central obesity had an OR of 7.5 (p < 0.01), which was far ahead of other individual risk factors (ORs ≤ 2.7). Taking MetS component count as a potential risk factor in model 2, Table 4 shows that adults having single and double MetS component had respectively a 2.8-fold (95% CI, 1.6-4.7) and 7.3-fold (95% CI, 4.3-12.6) increased risk of progression to MetS, compared with adults initially having zero MetS component count. In model 3 (Table 5), the entire single and two MetS component-carrying statuses, totally fifteen isolated assortments were introduced. Table 5 indicates all the ORs (from 59.6 to 3.9, all p < 0.05) of two-component combinations were greater than the ORs of single components except for COB-alone (OR, 9.1, p < 0.01). Among the two-component combinations, synergistic effects on MetS development were found in following combinations: HBP-plus-Low-HDL with OR of 11.7(p < 0.01), and HBP-plus-hyperglycemia with OR of 7.9 (p < 0.01) for MetS development compared with the adults having zero MetS component count, synergistic indexes were 4.7 (95% CI, 2.1-10.9) and 2.7 (95% CI, 1.2-6.4), respectively.

**Table 3:** Model 1, adjusted risks for metabolic syndrome (MetS), conventionally, individual MetS-components are not separated apart

Baseline potential risk factors*	Adjusted-OR**	95%CI	*p*
MetS component			
COB	7.5	4.0 - 14.2	<0.01
Hyper-TG	2.4	1.5 - 3.7	<0.01
Hyper-GL	2.2	1.4 - 3.3	<0.01
Low-HDL	2.7	1.8 - 4.0	<0.01
HBP	2.5	1.6 - 3.9	<0.01
Hyperuricemia	1.0	0.6 - 1.6	0.98
Sonographic fatty liver	2.3	1.6 - 3.5	<0.01
Elevated alanine aminotransferase	1.3	0.8 - 2.1	0.36
Elevated γ-glutamil transferase	2.0	1.3 - 3.1	<0.01
Hyperinsulinemia	1.7	1.1 - 2.6	<0.01

* Abbreviations: Hyper-GL, hyperglycemia; Low-HDL, Low-HDL cholesterolemia; HBP, High blood pressure; Hyper-TG, hypertriglyceridemia; COB, central obesity

** Adjusted variables were age and categorical variables including gender, smoking, diet habits, drinking, exercise status, sonographic liver enzymes, fatty liver, hepatovirus infections, hyperinsulinemia and hyperuricemia.

**Table 4:** Model 2, adjusted risks for metabolic syndrome (MetS) using MetS-component count as potential risk factors

Baseline potential risk factors	Adjusted-OR*	95%CI	*p*
MetS component count			
Adults having a single MetS component	2.8	1.6 - 4.7	<0.01
Adults having two MetS components	7.3	4.3 - 12.6	<0.01
Hyperuricemia	1.0	0.6 - 1.6	0.99
Sonographic fatty liver	2.4	1.6 - 3.6	<0.01
Elevated alanine aminotransferase	1.3	0.8 - 2.1	0.37
Elevated γ-glutamyl transferase	2.0	1.2 - 3.1	<0.01
Hyperinsulinemia	1.8	1.2 - 2.6	<0.01

* Adjusted variables were age and categorical variables including gender, smoking, diet habits, drinking, exercise status, liver enzymes, sonographic fatty liver, hepatovirus infections, hyperinsulinemia and hyperuricemia.

**Table 5:** Model 3, adjusted risks for metabolic syndrome (MetS) calculated using each MetS component and their combinations as potential risk factors

Baseline MetS components and combinations*	Adjusted-OR**	95%CI	Overall (N = 1384) *P*	SI^†^	95%CI
Adults having single MetS component at baseline	*NA*^‡^	-	-	-	-
COB alone	9.1	2.8 - 29.9	<0.01	*one risk alone*	-
Hyper-TG alone	3.4	1.5 - 7.7	<0.01	*one risk alone*	-
Hyper-GL alone	3.0	1.5 - 6.0	<0.01	*one risk alone*	-
Low-HDL alone	2.9	1.4 - 5.8	<0.01	*one risk alone*	-
HBP alone	1.4	0.6 - 3.4	0.44	*one risk alone*	-

Adults having two MetS components at baseline	*NA*				
COB-plus-Hyper-TG	59.6	5.8 - 611.5	<0.01	7.6	0.8 - 74.5
COB-plus-Low-HDL	24.6	7.7 - 78.2	<0.01	2.8	0.8 - 9.2
COB-plus-HBP	14.5	3.6 - 59.1	<0.01	2.0	0.5 - 8.5
HBP-plus-Low-HDL	11.7	5.1 - 27.0	<0.01	**4.7**	**2.1 - 10.9**
HBP-plus-Hyper-GL	7.9	3.5 - 18.1	<0.01	**2.7**	**1.2 - 6.4**
COB-plus-Hyper-GL	6.7	1.2 - 38.7	<0.05	0.8	0.1 - 5.2
HBP-plus-Hyper-TG	5.8	2.2 - 15.4	<0.01	1.6	0.6 - 4.3
Low-HDL-plus-Hyper-TG	5.7	2.7 - 12.3	<0.01	1.6	0.7 - 3.3
Hyper-GL-plus-Hyper-TG	4.1	1.5 - 11.1	<0.01	1.6	0.6 - 4.4
Hyper-GL-plus-Low-HDL	3.9	1.6 - 9.8	<0.01	1.1	0.4 - 2.9

* Abbreviations: Hyper-GL, hyperglycemia; Low-HDL, Low-HDL cholesterolemia; HBP, high blood pressure; Hyper-TG, hypertriglyceridemia; COB, central obesity.;.

** Adjusted variables were age and categorical variables including gender, smoking, diet habits, drinking, exercise status, liver enzymes, sonographic fatty liver, hepatovirus infections, hyperinsulinemia and hyperuricemia.

^† ^SI, synergistic index, SI value exceeding 1.0 indicates the presence of synergistic interaction.

^‡ ^NA: Not analyzed in this model, analyzed and shown in Table 4, Model 2.

SI and 95%CI in bold are considered to be statistically significant.

## Discussion and Conclusions

At the baseline screening for MetS among our middle-aged working population, 84% (1384 from 1640) of the employees did not fulfill MetS criteria. However, within five years, a considerable number (13%, 175 from 1384) of the middle-aged workers fulfilled criteria of MetS. The incidence of MetS in our middle-aged working population was as high as reported in a survey for Chinese middle-aged adults [18]. Risk evaluations for MetS development in general population had attracted attentions of public health professionals [4-7]; furthermore, risk assessments for MetS development are also necessary for all middle-aged adults, including the early-middle-aged adults potentially at risk, but not yet fulfilling MetS criteria at screening. In a previous worksite MetS research, applying a health promotion program for high-risk persons, Richard V. and colleagues obtained decreased total health risk as well as markedly reduced medical claim costs; and finally, over half of all baseline high-risk persons were converted to low-risk statuses [19]. In terms of health management, our results can help use the readymade screening records to identify high-risk adults and start intervention as soon as possible.

Initially, over half of the adults from our sample population had at least one MetS component (58.6%, Table 1; 36.9% with single and 21.7% with two components, Table 2). Our follow-up survey demonstrated a significant correlation between the baseline MetS component count and MetS development. The middle-aged adults having one or two MetS components had respectively 2.8-fold and 7.3-fold increased risks for developing MetS within five years, versus the adults having zero MetS component count (Table 4). Some cardiovascular disease (CVD)-related surveillance studies had similar findings: the MetS component count has been associated with CVD occurrence[20], mortalities[21] and important surrogate markers[22]. Furthermore, Schultz and Edington demonstrated that changes in MetS component count were significantly related to the medical costs for workforces[23]. As a relatively simple measurement, we suggest using the MetS component count as a fundamental item for risk assessment in MetS management programs.

Throughout our analyses, central obesity was the strongest predictor for MetS development out of all risk factors (Table 3 and Table 5), regardless of its combinations with any other individual risk factors (Table 5). Since the "yo-yo" effects appeared in many trials for weight reduction[24], our observation support that prevention for obesity should be strongly prioritized for middle-aged adults in terms of MetS prevention[25]. Previous investigations and our current analyses both agreed that elevated γ-GT[26], hyperinsulinemia[27] and sonographic fatty liver[28] are all acceptable predictors for MetS development. However, according to our multivariate analysis (Table 3, Table 4), the ORs of the risk factors mentioned above were not significantly greater than ORs of MetS components. On the other hand, all measurements of MetS components are available in most medical settings[29], in contrast to many equipment-requiring image examinations or technique-dependent measurements of surrogate markers [30-33]. Such a relatively accessible program capable of making its contributions simultaneously to abnormality screening and effective risk assessments, MetS screening program naturally has an important aspect of public health promotion.

Our component-component interaction analyses demonstrated several significant compound effects of MetS components on MetS development. In our two-component analyses, the middle-aged adults simultaneously having HBP and any other MetS component were at a dramatically increased risk for MetS development (model 3, Table 5, lower rows). The results of the analyses for MetS development were relatively close to clinical observations: together with other risk factors, hypertension had aggravating effects on the cardiovascular risks [34]. In addition, high blood pressure clustered with raised blood glucose or low HDL cholesterol were the riskiest combinations for arterial disease or premature death [35,36]. Health managers should emphasize blood pressure control in workplace-based MetS management programs because of its high abnormality rate among the middle-aged population and its synergistic interactions with other risk factors.

The major limitation of this observational investigation is that we did not take into consideration potential treatment effects. For some persons with one or two MetS components, it was possible that they could have utilized corrective options including lifestyles changes[37] or medications such as antihypertensive [38] or lipids lowering agents[39]. Those actions might lead to protective effects[40], and therefore, the true risk of MetS development might be underestimated in this early-middle-aged population. Naturally, this is a workplace follow-up survey for relatively healthy workers. Thus, the application of our conclusions to the general population should be cautious. On the other hand, statistically, our findings of MetS component combinations were based on low case numbers, which might have resulted in a low number bias. Thus, larger sample population studies are needed in the future.

In sum, a workplace-based MetS screening program simultaneously allows for finding out cases and for assessing risk of MetS development. MetS component count and combination can be used in predicting MetS development for participants potentially at risk. For MetS prevention, any increase in the number of MetS components should be avoided. Since central obesity plays a pivotal role and high blood pressure synergistically potentiates other risk factors toward MetS development, efforts to address these two easily measurable risk factors must be prioritized in workplace MetS management programs.

## Abbreviations

COB: central obesity; HBP: high blood pressure; Hyper-GL: hyperglycemia; Hyper-TG: hypertriglyceridemia; Low-HDL: Low-HDL cholesterolemia; SI: synergistic index;

## Competing interests

The authors declare that they have no competing interests.

## Authors' contributions

YCL collected and analysis data; YCL, SHL and JDC interpreted data; YC L drafted the article; SHL and JDC revised critically for important intellectual content; PCC final approved the version to be published. All authors read and approved the final manuscript.

## Pre-publication history

The pre-publication history for this paper can be accessed here:

http://www.biomedcentral.com/1471-2458/10/747/prepub
